# Complete Genome Sequence of a Phapecoctavirus Isolated from a Pigeon Cloacal Swab Sample

**DOI:** 10.1128/MRA.01471-20

**Published:** 2021-02-04

**Authors:** Anthony Khalifeh, Simona Kraberger, Daria Dziewulska, Tomasz Stenzel, Arvind Varsani

**Affiliations:** a Biodesign Center for Fundamental and Applied Microbiomics, Center for Evolution and Medicine, School of Life Sciences, Arizona State University, Tempe, Arizona, USA; b Department of Poultry Diseases, Faculty of Veterinary Medicine, University of Warmia and Mazury, Olsztyn, Poland; c Structural Biology Research Unit, Department of Integrative Biomedical Sciences, University of Cape Town, Observatory, Cape Town, South Africa; DOE Joint Genome Institute

## Abstract

The complete genome sequence of a bacteriophage in the genus *Phapecoctavirus* (family *Myoviridae*) isolated from a cloacal swab specimen from a domestic pigeon (*Columba livia* f. *domestica*) was identified using a high-throughput sequencing approach. The genome is 150,892 bp with a GC content of 39.1%, containing 269 open reading frames and 11 tRNA genes.

## ANNOUNCEMENT

Over the past decade, with the use of high-throughput sequencing, a plethora of known and novel viral sequences have been identified from a variety of sample types. For avian samples, cloacal swab specimens have been used for the identification of various pathogenic viruses, such as circoviruses, flaviviruses, gyroviruses, orthomyxoviruses, papillomaviruses, paramyxoviruses, and polyomaviruses. Additionally, numerous bacteriophages have been identified, including those classified in the *Myoviridae* family (1). Myoviruses are double-stranded DNA (dsDNA) viruses that have contractile tails (2). Identifying new viral genomes helps provide insights into viral diversity, evolution, and putative hosts in the case of bacteriophages. Here, we describe a new member of the *Phapecoctavirus* genus (family *Myoviridae*) that was identified in a cloacal swab specimen collected from an 8-week-old racing/carrier domestic pigeon (*Columba livia* f. *domestica*) from an amateur/hobby pigeon facility located in northern Poland (Samolubie, Bartoszyckie County, Warmińskpo-Mazurskie voivodeship; GPS coordinates, 54°11′02.1″N, 20°43′45.2″E).

Viral DNA was extracted from a pigeon cloacal swab specimen using the High Pure viral nucleic acid kit (Roche Diagnostics, USA). Viral DNA was amplified using rolling circular amplification (RCA) with the TempliPhi 2000 kit (GE Healthcare, USA). The resulting RCA DNA was used to generate 2 × 150-bp libraries at BGI (Hong Kong) using their DNBseq normal DNA library option, and the libraries were sequenced on their BGIseq sequencer. The resulting reads (11,610,789 read pairs) were quality trimmed using Trimmomatic v0.39 (3), and the trimmed reads were *de novo* assembled using metaSPAdes v3.12.0 (4). Contigs of >1,000 nucleotides (nt) were analyzed against an NCBI RefSeq (5) viral protein sequence database, and bacteriophages were identified using VirSorter (6). All tools were run with default parameters unless otherwise specified. A circular contig (based on terminal redundancy) of 150,892 nt (GC content, 39.1%) was identified that is most closely related to viruses in the genus *Phapecoctavirus* (family *Myoviridae*). A total of 36,072 reads mapped to this bacteriophage genome with a mean coverage of 16×. RASTtk (7) was used to annotate this genome and predicted 269 open reading frames (varying in size from 96 to 3,333 nt) and 11 tRNA genes (Arg, Asn, Gln, Gly, Ile, Met, Met, Pro, Ser, Thr, and Tyr). We tentatively name this virus dompiswa virus (domestic pigeon swab-associated virus).

**FIG 1:**
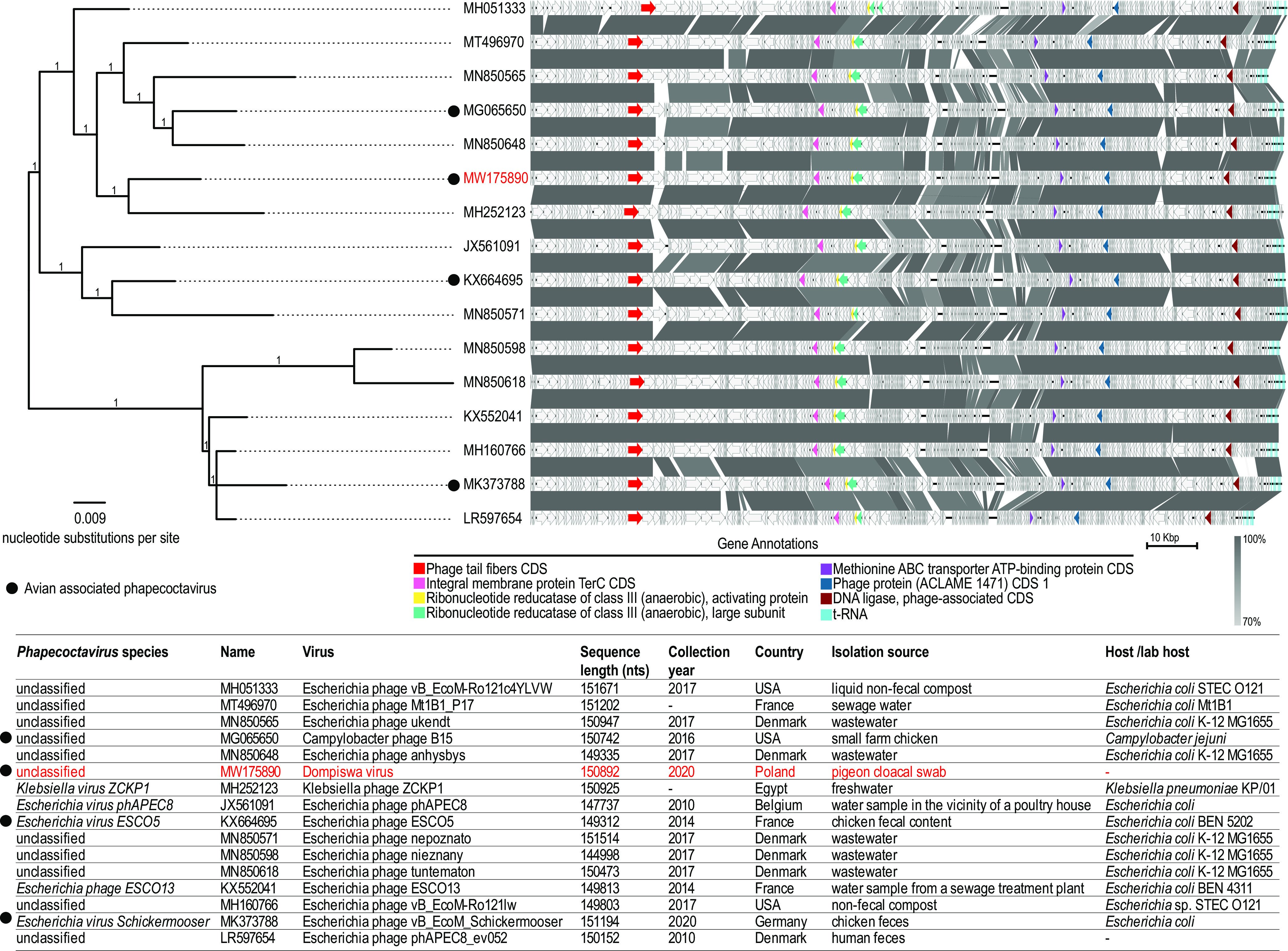
Phylogenetic analysis of phapecoctavirus genomes (*n* = 16). The accession number of the dompiswa virus and associated information are highlighted in red. Comparisons of phapecoctavirus genomes are shown to the right of the phylogeny. Gene annotations are highlighted to show relative gene placement along the genomes. Gray boxes between sequences indicate similarity of regions based on BLASTn analysis. CDS, coding sequence.

All related genomes belonging to the genus *Phapecoctavirus* (*n* = 15) were downloaded from GenBank (on 12 November 2020). The genomes were linearized at the end of the cluster of tRNA genes and aligned with MAFFT (8), and the resulting alignment was used to infer a maximum likelihood phylogenetic tree with PhyML (9) using the WAG+G+I nucleotide substitution model (determined to be the best-fit model using ModelTest [10]). Branches with approximate likelihood ratio test support of <0.8 were collapsed using TreeGraph 2 (11) and midpoint rooted. Phylogenetically, dompiswa virus is most closely related to *Klebsiella* phage ZCKP1 (GenBank accession number MH252123), isolated from freshwater (12), sharing 92.3% intergenomic distance determined using VIRIDIC (13), and it clusters with other unclassified phages (GenBank accession numbers MH051333, MT496970, MN850565, MG065650, and MN850648) (Fig. 1).

Five of the 16 phapecoctaviruses have been identified from avian fecal sources, and according to culture-based laboratory approaches, 14 have been shown to infect enterobacteria (Campylobacter jejuni, Escherichia coli, or Klebsiella pneumoniae). Therefore, it is likely that dompiswa virus infects enterobacteria, but this needs to be confirmed.

### Data availability.

This genome sequence has been deposited in GenBank under the accession number MW175890, and mapped short reads have been deposited in the SRA under the accession number SRR12914778.
